# Discrimination of Milk Freshness Based on Synchronous Two-Dimensional Visible/Near-Infrared Correlation Spectroscopy Coupled with Chemometrics

**DOI:** 10.3390/molecules28155728

**Published:** 2023-07-28

**Authors:** Dan Peng, Rui Xu, Qi Zhou, Jinxia Yue, Min Su, Shaoshuai Zheng, Jun Li

**Affiliations:** 1 College of Food Science and Engineering, Henan University of Technology, Zhengzhou 450001, China; 2021920028@stu.haut.edu.cn (Q.Z.); 2021920008@stu.haut.edu.cn (M.S.); 2022920031@stu.haut.edu.cn (S.Z.); 2 School of International Education, Henan University of Technology, Zhengzhou 450001, China; 201920010112@stu.haut.edu.cn; 3 Shandong Yuxin Bio-Tech Co., Ltd., Binzhou 256600, China; 18236958515@163.com

**Keywords:** milk, freshness, Vis/NIR, two-dimensional correlation spectra, chemometrics

## Abstract

Milk is one of the preferred beverages in modern healthy diets, and its freshness is of great significance for product sales and applications. By combining the two-dimensional (2D) correlation spectroscopy technique and chemometrics, a new method based on visible/near-infrared (Vis/NIR) spectroscopy was proposed to discriminate the freshness of milk. To clarify the relationship be-tween the freshness of milk and the spectra, the changes in the physicochemical indicators of milk during storage were analyzed as well as the Vis/NIR spectra and the 2D-Vis/NIR correlation spectra. The threshold-value method, linear discriminant analysis (LDA) method, and support vector machine (SVM) method were used to construct the discriminant models of milk freshness, and the parameters of the SVM-based models were optimized by the grid search method and particle swarm optimization algorithm. The results showed that with the prolongation of storage time, the absorbance of the Vis/NIR spectra of milk gradually increased, and the intensity of autocorrelation peaks and cross peaks in synchronous 2D-Vis/NIR spectra also increased significantly. Compared with the SVM-based models using Vis/NIR spectra, the SVM-based model using 2D-Vis/NIR spectra had a >15% higher prediction accuracy. Under the same conditions, the prediction performances of the SVM-based models were better than those of the threshold-value-based or LDA-based models. In addition, the accuracy rate of the SVM-based model using the synchronous 2D-Vis/NIR autocorrelation spectra was >97%. This work indicates that the 2D-Vis/NIR correlation spectra coupled with chemometrics is a great pattern to rapidly discriminate the freshness of milk, which provides technical support for improving the evaluation system of milk quality and maintaining the safety of milk product quality.

## 1. Introduction

Milk is a natural food with high nutritional values. It is rich in lipids, proteins, lactose, vitamins, minerals, and other bioactive nutrients [1,2], and is thus deeply loved by consumers. Milk, however, is very perishable. Particularly, the improper storage or lack of a complete cold chain system in collection, processing, transportation, and sales leads to lactose being fermented to produce acids, increasing the total number of colonies, causing fat oxidation and rancidity, and largely reducing its nutritional and edible values [3,4]. Different solutions have been developed to delay the occurrence of milk spoilage, such as thermal treatment, special package, refrigeration, etc. [5]. However, the changes in environmental conditions or damaged packages still cause milk to spoil before shelf life. Around 128 million tons of milk are wasted globally every year [6]. Therefore, monitoring the freshness of milk is crucial to ensure the safety of dairy products and the health of consumers.

At present, there are two main methods for the identification of milk freshness. One is based on the evaluation of microbial growth, such as total aerobic bacteria (TAB), psychrotrophic bacteria (PCB), and lactic acid bacteria (LAB) [7]. The method can characterize the internal freshness of milk, but requires skilled technicians, analytical equipment, and a long measurement period. Hwang et al. [8] found that the electrical conductivity freshness index can predict the growth of TAB, PCB, and LAB in milk. However, the electrical conductivity method is limited to specific contaminants and temperature ranges, and the model works well at 20 °C, which is significantly higher than the typical refrigeration temperature. The other is to judge the freshness by the internal composition, odor, or physical and chemical indices of the milk, such as acidity, lactose, fatty acid, and pH value [9,10,11]. Using the fluorescein isothiocyanate (FITC)/FITC-dextran (FITC-dex) and Tris (2,2′-bipyridyl) dichloro ruthenium (II) hexahydrate (Ru-bpy), Choudhary et al. [12] developed the solution phase and immobilized phase pH sensors to monitor the changes in milk quality. This approach is successful to a certain extent. However, the safety of the materials used for sensor preparation needs to be further evaluated. In addition, headspace odor sensing was also reported to determine milk freshness or spoilage [13,14,15,16,17]. However, the volatile inventories reported by different researchers vary largely depending on the differences in milk freshness and spoilage conditions, and there is not yet a clear set of volatiles as reliable indicators for the freshness or spoiled state of milk. The instruments used to monitor milk freshness or spoilage must have sufficient sensitivity, specificity, and a detection limit. Liquid chromatography-high resolution mass spectrometry has been reported for the classification of non-fresh milk [18]. Although it offers high-level analytical accuracy, the expensiveness of instruments, the need for professional skills, and the high running costs make it economically unviable for routine milk monitoring.

In recent years, visible/near-infrared (Vis/NIR) spectroscopy has been recognized as a rapid, non-destructive, and environmentally friendly detection technique and has been applied in the evaluation of food freshness. Zhang et al. [19] used Vis/NIR spectroscopy to quickly identify the freshness of mutton. Nakajima et al. [20] used Vis/NIR spectroscopy to evaluate the freshness of stored cabbage. Kuroki et al. [21] used Vis/NIR spectroscopy to evaluate the freshness of spinach leaves under low-oxygen conditions. Vis/NIR spectroscopy was also used to evaluate the freshness of protein and pork [22,23]. However, there are few reports on the detection of milk freshness by Vis/NIR spectroscopy. This is because some problems need to be overcome when using the Vis/NIR spectroscopy technique to detect the quality of milk and its dairy products, such as sensitivity. In addition to absorbing light, milk also has strong scattering properties [24], which makes it extremely difficult to extract the useful information from the spectrum. Moreover, the wide spectral band and overlapping of the characteristic absorption peaks further increase the difficulty in extracting the useful information. In this work, consequently, the 2D correlation analysis technique was introduced into the Vis/NIR spectroscopy of milk to improve the extraction of the useful information from its spectra.

The emergence and rapid development of chemometrics provide a theoretical tool for the analysis of complex data. Chemometrics is the science of extracting the chemical information from data using mathematical and statistical tools [25]. In recent years, various analytical methods (chromatography, mass spectrometry, spectrometry, etc.) coupled with chemometrics have been used in the detection of dairy products, such as the geographic origin of cheese [26], quality identification of milk powder [27], milk adulteration [22], and others. In general, chemometric methods can be classified into qualitative and quantitative, or linear and nonlinear methods. Common linear methods include multiple linear regression (MLR), linear discriminant analysis (LDA), partial least square (PLS), etc., while the most commonly used nonlinear methods are artificial neural network (ANN), support vector machine (SVM), etc. In this study, the discrimination model of milk freshness was established by linear discriminant analysis (LDA) and support vector machine (SVM). Spectral changes during milk storage were studied to determine the relationship between spectral peaks and freshness. Different modeling methods were compared to evaluate the capacity of the 2D-Vis/NIR correlation spectroscopy technique in the detection of milk freshness.

## 2. Results and Discussion

### 2.1. Analysis of Physical and Chemical Indices of Milk

**Figure 1:**
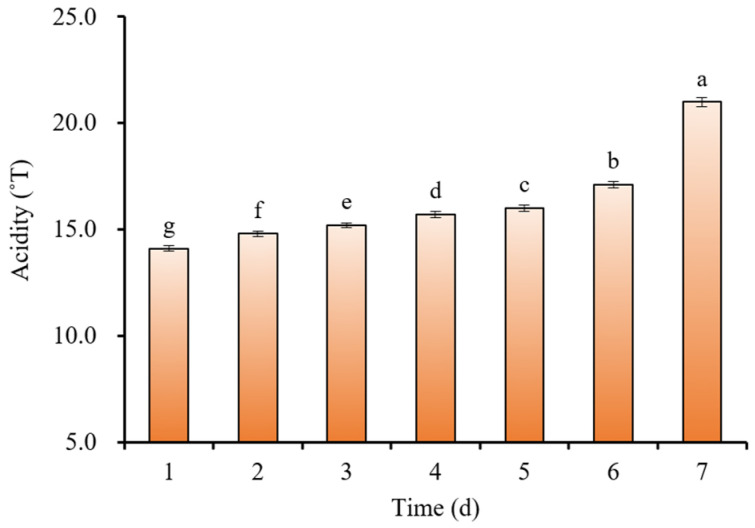
Change in milk acidity during storage (different lowercases indicated the significant difference (*p* < 0.05).

The freshness of milk samples can be characterized by physicochemical indices [26], and the changes in the physicochemical indices of milk during low-temperature storage are shown in Table 1. The relative density and fat and protein contents of milk changed minimally with the coefficients of variation (CV) of <0.02 during storage. The protein and fat contents were still within the range of the National Standard GB 19301-2010 [28] during 6 days of storage. However, with the extension of storage time, the acidity of milk increased by 6.9 °T with a CV of 0.1408; the lactose content in milk decreased by 0.32% with a CV of 0.0217. The acidity of milk was initially 14.1 °T (Figure 1), and increased gradually within the first 4 days and rapidly from the 5th day onwards. The acidity of milk reached 21 °T after a storage time of 6 days, which exceeded the acidity requirement (12~18 °T) regulated by the National Standard GB 19301-2010. Sensorily, milk exhibited signs of internal coagulation in the form of lumps, which indicates that the milk had deteriorated and was not suitable for consumption. Within 6 days of low-temperature storage, the changing degree of each physicochemical index of milk followed the order: acidity > lactose > other indicators (relative density and fat and protein contents). Thus, acidity can better characterize the freshness of milk and was used as the characteristic indicator to classify the milk samples into three types: fresh A (13~15 °T), sub-fresh B (15~18 °T), and spoiled C (>18 °T).

### 2.2. Analysis of Vis/NIR Spectra and Synchronous 2D-Vis/NIR Spectra of Milk

**Figure 2:**
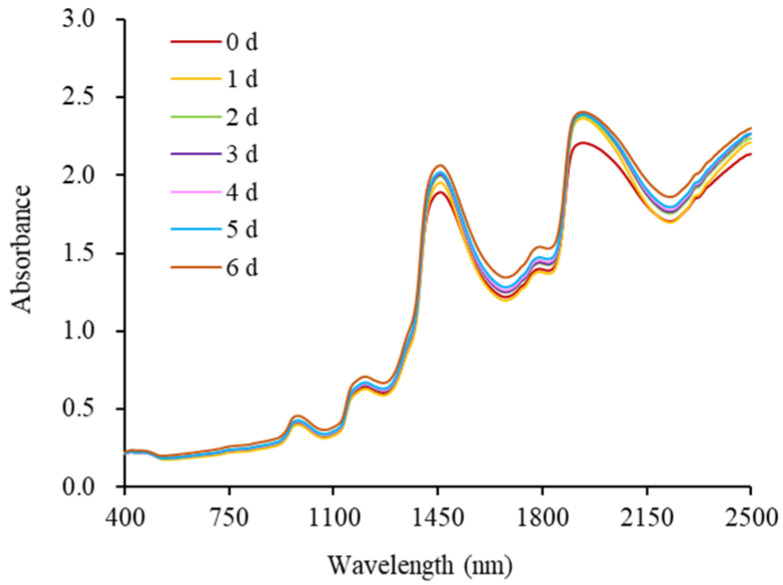
Vis/NIR spectra of milk during storage.

The Vis/NIR spectra of milk samples with different storage times are shown in Figure 2. Regardless of freshness, all milk samples had a roughly similar overall trend in the Vis/NIR spectra. With the increase in storage time, significant differences were observed in the wavelength ranges of 1400~1508 nm and 1882~2100 nm, which is attributed to the changes in the internal tissue structures and component contents of milk. The absorption peaks at 1450 nm and 1940 nm were associated with water and were the first overtone and combination bands of O-H residue, respectively [29]. The absorption bands corresponding to the fat and fatty acid contents were located at 1210, 1790, and 2300 nm, respectively [30]. The absorption peaks at 2050 and 2180 nm indicated the protein content. The characteristic absorptions of lactose and lactic acid were located at 1194, 1560~1750, and 2094 nm [31]. In addition, the absorption peaks of carotenoids in milk were located at 460 nm [32].

Although there were some differences in the one-dimensional Vis/NIR spectra of milk at different storage times (freshness), the variations were relatively small. Particularly, the spectra of sub-fresh milk samples and spoiled milk samples were difficult to be distinguished. Thus, the 2D correlation spectroscopy technique was introduced into the study to improve the resolution of Vis/NIR spectra.

**Figure 3:**
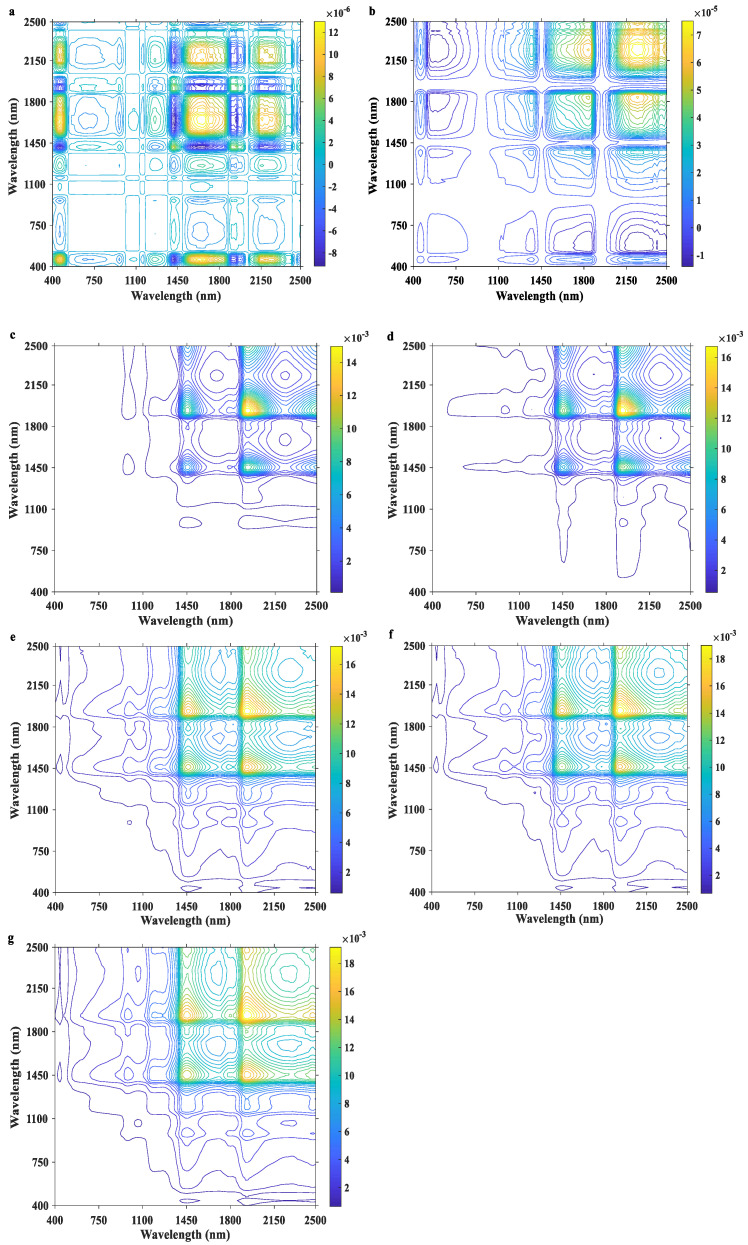
Synchronous 2D-Vis/NIR spectra of milk at different storage times ((**a**) 0 day; (**b**) 1 day; (**c**) 2 days; (**d**) 3 days; (**e**) 4 days; (**f**) 5 days; (**g**) 6 days).

The synchronous 2D-Vis/NIR spectra of milk are shown in Figure 3. Significant differences were observed in the 2D correlation spectra of milk with different storage times. Ideally, a set of n × n synchronous spectral matrices with zero values should be obtained after the 2D correlation analysis of fresh milk spectra and reference spectra. However, the synchronous spectral matrix of fresh milk was not exactly zero due to various factors, such as the samples themselves and the stability of the instrument employed. When the storage time was less than 24 h, neither obvious autocorrelation peaks nor cross peaks were observed in the synchronous 2D-Vis/NIR spectra of milk, and their peak values were all <0.0001. When the storage time was 48 h, two strong autocorrelation peaks and three positive cross peaks appeared in the synchronous spectra, and the cross peaks were located at (1450, 1940), (1450, 2498), and (1940, 2498) with values of >0.01, >0.006, and >0.01, respectively. The intensities of the autocorrelation peaks (1450 nm) and cross peaks (1450, 1940) in the synchronous spectra increased significantly with the extended storage time and enhanced all >10^4^ times after 6 days of storage time. This is consistent with the results from the physicochemical indicators of spoiled milk (Table 1 and Figure 1).

**Figure 4:**
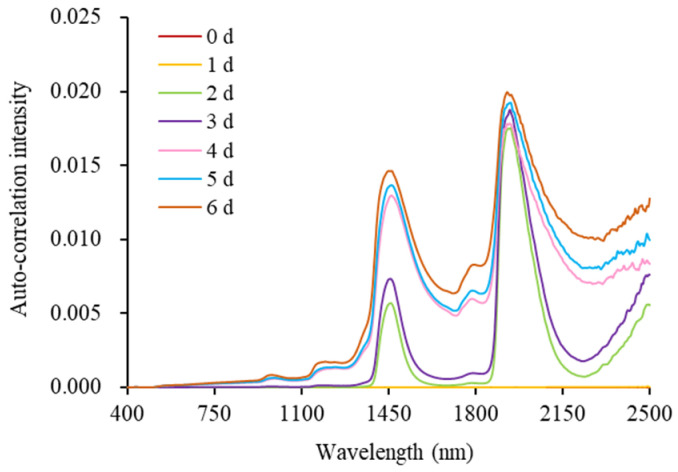
Synchronous 2D-Vis/NIR autocorrelation spectra of milk during storage.

The synchronous 2D-Vis/NIR autocorrelation spectra of milk under different storage conditions are shown in Figure 4. The intensities of the autocorrelation peaks at 1194, 1450, 1790, and 1940 nm showed a significant increasing trend with the prolongation of storage time, and the information contained at these wavelength positions were all related to the freshness indices of milk. Based on the changes in each index during milk storage and the results in Figure 2, it was initially determined that the autocorrelation peaks at 1450 and 1940 nm were attributed to water, the autocorrelation peak at 1194 nm was related to lactose and lactic acid, and the autocorrelation peak at 1790 nm reflected the fat-related information. Compared with the traditional Vis/NIR spectra (Figure 2), the synchronous 2D Vis/NIR autocorrelation spectroscopy can more clearly show the relationship between the internal quality changes in milk and different spectral bands under different storage times. In summary, the three types of milk can be directly distinguished based on the differences in synchronous 2D-Vis/NIR spectra. Therefore, the 2D-Vis/NIR technique has potential applications as a visualization method to explore the freshness of milk.

### 2.3. Milk Freshness Identification

#### 2.3.1. Threshold-Value Method

**Figure 5:**
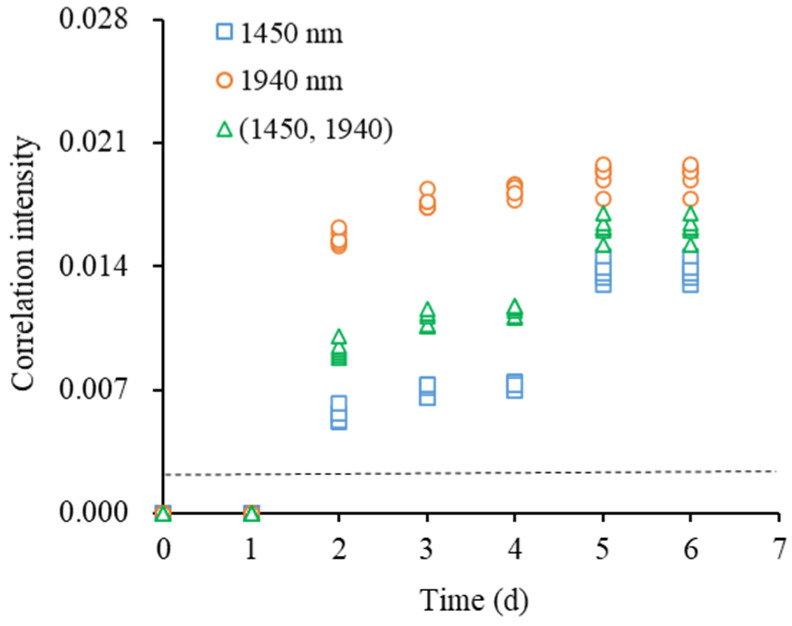
Discriminant results with different feature peaks using threshold-value method (The correlation intensity of the feature peaks of samples indicated the milk was fresh when being below the dashed line and the milk was non-fresh when being above the dashed line).

In the threshold-value method, the freshness of milk was judged by the values of the peaks in the synchronous 2D correlation spectra. The standard deviations of the autocorrelation peaks of fresh milk at 1450 and 1940 nm and the cross peaks at (1450, 1940) were less than 3.0 × 10^−5^ (2.87 × 10^−5^, 1.04 × 10^−5^, and 1.29 × 10^−5^), indicating that the dispersions of peak values at the same location were small. Therefore, the peak values of the above three locations were taken as the discrimination threshold, as shown in Figure 5. Using the three values of peaks, the identification accuracy for fresh milk was 100%. In particular, the autocorrelation peak at 1940 nm between fresh and non-fresh milk was more differentiated from the discrimination threshold, while the differentiation was small for sub-fresh and spoiled milk samples. In contrast, the peaks at 1940 nm and (1450, 1940) had a good linear relationship with storage time, and their correlation coefficients (R^2^) were 0.92 and 0.89, which indicates that the storage time (freshness) of milk can be roughly predicted based on these two peaks. However, as the storage time increased, the standard deviations of the peak values from different samples increased, reducing the prediction accuracy of milk freshness. Therefore, the threshold-value method can be used to only simply identify the fresh milk in some simple scenarios.

#### 2.3.2. LDA Identification Analysis

The spectral data contain not only the useful information of analytes, but also irrelevant information, such as noise, background, stray light, etc. To eliminate the influences of irrelevant information, the first derivative (1st), multiplicative scatter correction (MSC), and standard normal variate (SNV) methods were used to preprocess the Vis/NIR and 2D-Vis/NIR spectra, followed by establishing milk classification models by the LDA method with linear as the discriminant function (Table 2). The prediction accuracy of the calibration set of the models improved slightly after the spectra were treated by MSC or SNV, while the robustness of the models decreased significantly after the 1st treatment, and the prediction accuracy of the prediction set reduced by >45%. This may be because the noise of the original data was amplified after the 1st treatment, increasing the interference information and decreasing the spectral signal-to-noise ratio. Considering the prediction accuracy of the model and easy-to-use, original data rather than pretreated data were directly used for following studies.

**Figure 6:**
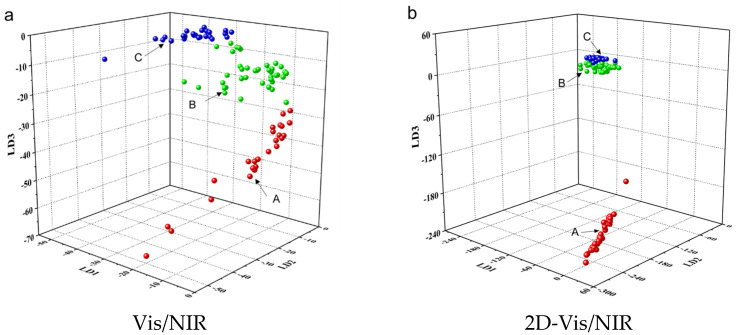
Validation results of LDA model (A: fresh milk, B: sub-fresh milk, and C: spoiled milk).

After comparing the modeling performances of the Vis/NIR and 2D-Vis/NIR datasets, it was found that the 2D correlation spectroscopy technique led the samples in the new subspace to have relatively close intra-class distances and relatively far inter-class distances (Figure 6), especially between the fresh milk samples and the spoiled milk samples. The performances of the 2D-Vis/NIR-based models were better than those of the Vis/NIR-based models, with a prediction accuracy of 100%, 86.7%, and 100% for fresh milk (A), sub-fresh milk (B), and spoiled milk (C), respectively. The overall improvement of 3% from the 2D-Vis/NIR-based models was obtained in comparison with that of the Vis/NIR-based models. Since milk is a very complex medium [33], there is no ideal linear relationship between the spectra and physicochemical indicators of milk, making the LDA-based models unable to completely distinguish sub-fresh and spoiled milk. Jin et al. [34] used LDA and ANN to construct a discrimination model for recovered and fresh milk, and the prediction performance of the nonlinear ANN-based model (99.6%) was better than that of the LDA-based model (94.9%), which also further illustrates the unsatisfactory applicability of LDA for nonlinear problems with complex systems.

#### 2.3.3. SVM Identification Analysis

**Figure 7:**
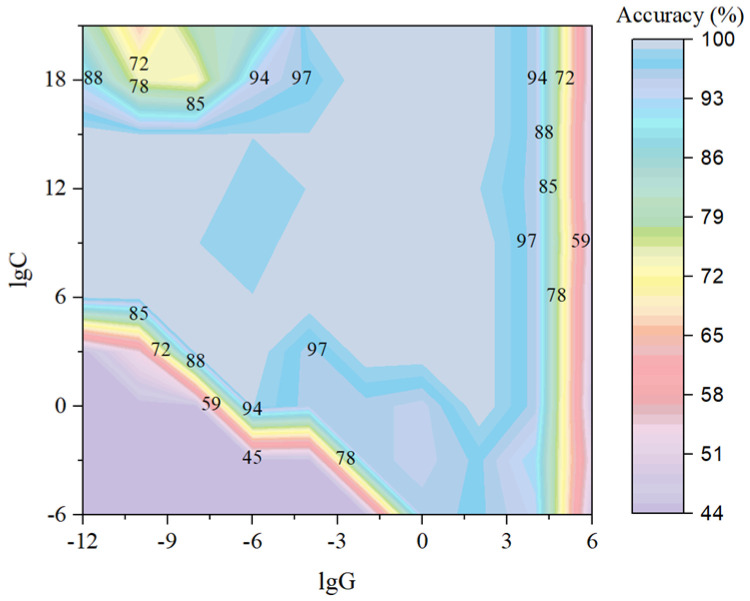
Optimization process of SVM-based model parameters [C, G] using 2D-Vis/NIR data (RBF as kernel function).

Four kernel functions (linear, polynomial, RBF, and sigmoid) were used to construct SVM-based models using the Vis/NIR and 2D-Vis/NIR spectra of milk, and the optimum model parameters were selected by the grid search method and particle swarm optimization. The results are shown in Figure 7 and Table 3. The cross-validation method was used to evaluate the performance of the SVM-based models with accuracy as the evaluation indicator. Taking the modeling process of the 2D-Vis/NIR spectral data of milk as an example, the optimized region of parameters was determined by a grid search with large steps, and then the best combination of [C, G] was found by the particle swarm algorithm when the RBF kernel was selected. Too large or too small values of C and G will cause the overfitting or underfitting phenomenon of the model. After the parameter optimization, when [C, G] was [7.74 × 10^3^, 3.59], the kernel function was RBF, the number of support vectors was 6, and the accuracy of the SVM-based model was 100%. Similarly, the parameters of other SVM-based models were optimized, and the results are shown in Table 3.

All SVM-based models can discriminate fresh milk with an accuracy of up to 100% (Table 3), which is the same prediction performance as the threshold-value-based and LDA-based models. The performances of SVM-based models using 2D-Vis/NIR spectra data were significantly better than those using Vis/NIR spectra data, and the prediction accuracy of the calibration sets were all greater than 97%. Although SVM-based models using 2D-Vis/NIR data had significantly fewer support vectors than SVM-based models using Vis/NIR data, the prediction abilities improved for the three types of milk, which indicates that 2D correlation spectroscopy can effectively extract the useful information and enhance the robustness of the models. The SVM-based model performed best when the kernel function was RBF, with 100% accuracy for all three types of milk, followed by linear, while the model with polynomial as the kernel function showed a relatively poor performance and had difficulty in distinguishing between the sub-fresh and spoiled milk samples.

**Figure 8:**
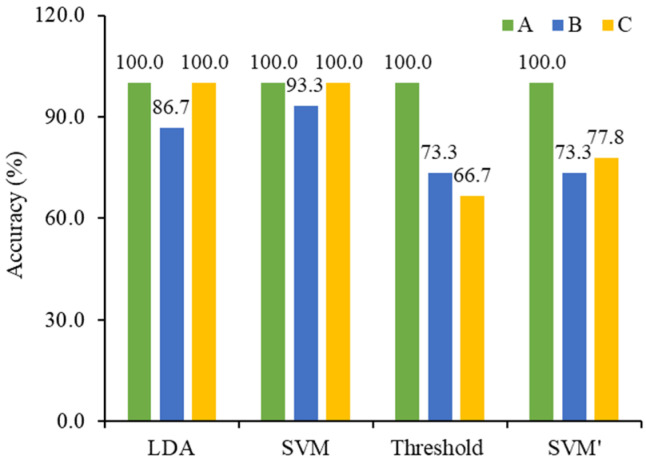
Prediction results of different models using 2D-Vis/NIR (for LDA, SVM, and threshold-value) data or Vis/NIR (for SVM’) data.

To verify the modeling performance, 33 unknown milk samples were selected as the prediction set and the 2D-Vis/NIR data were used as independent variables to predict the freshness of milk by SVM-based, LDA-based, and threshold-value-based models. After comparing the SVM-based models using Vis/NIR data, the results are shown in Figure 8. The prediction performances of the SVM-based models were better than those of the LDA-based and threshold-value-based models. For sample A (fresh milk), the prediction accuracies from the three methods were not much different and their accuracy rates were 100%. For sample B (sub-fresh milk) and sample C (spoiled milk), however, the prediction performances differed significantly. The SVM-based and LDA-based models using 2D-Vis/NIR data could accurately classify all samples C (spoiled milk), while their accuracy rates were 93.3% and 86.7% for sample B (sub-fresh milk). One sample B (sub-fresh milk) was wrongly identified as sample C (spoiled milk) by the SVM-based model, and two samples B (sub-fresh milk) were misjudged by the LDA-based model. The prediction performances of the threshold-value-based models were unsatisfactory, and the prediction accuracy was 73.3% and 66.7% for samples B (sub-fresh milk) and C (spoiled milk), respectively, with more than two erroneous judgements for both samples. The freshness monitoring of milk is related to food safety and consumers’ health, especially for the identification of spoiled milk. The identification of spoiled milk as sub-fresh milk here is unacceptable in this study. Compared with Vis/NIR, 2D-Vis/NIR improved the accuracy of SVM-based models by >15%. In summary, it is feasible to couple 2D-Vis/NIR spectroscopy with the SVM method for the prediction of the freshness of milk.

## 3. Materials and Methods

### 3.1. Sample Preparation

Fresh raw milk samples (10 types) were purchased at a local dairy factory (Zhengzhou, Henan, China) and the samples were immediately transported to the laboratory in a refrigerated box and placed in a 4 °C refrigerator for storage. The milk samples were stored for 6 days and subjected to Vis/NIR scanning and physicochemical analysis (fat, protein, lactose, acidity, etc.) every 12 h. Spectral information and physicochemical data were obtained from 130 milk samples with different degrees of freshness. Since the freshness of milk is closely related to its acidity, the milk samples were classified into fresh A (13~15 °T), sub-fresh B (15~18 °T), and spoiled C (>18 °T) according to their acidity status. The 130 milk samples were divided into two groups, and 97 samples were selected to form the calibration set and the remaining 33 samples formed the validation set.

### 3.2. Vis/NIR Collection

The diffuse reflectance spectra of the milk samples were acquired using a DS2500 NIR spectrometer (FOSS, Hillerød, Denmark) equipped with silicon (400~1100 nm) and lead sulfide (1100~2500 nm) as the detectors. The scanning range was 400~2500 nm with 32 scans, 2 nm resolution, and a detection temperature of 35 °C. The average of the three individual measurements was used for each sample.

### 3.3. Measurement of Milk Acidity

The acidity of the milk samples was quantified according to the National Standard method GB 5009.239-2016 [35]. Ten milliliters of milk samples were diluted with 20 mL of deionized water and then titrated with 0.1 mol/L NaOH solution using phenolphthalein as the indicator solution. The average of the three individual measurements was used for each sample.

### 3.4. Measurement of the Main Components of Milk

The protein content of the milk samples was determined by referring to the National Standard method GB/T 5009.5-2016 [36]. Fifteen grams of each sample were put into a digestion tube, followed by 0.4 g of copper sulfate, 6.0 g of potassium sulfate, and 20 mL of concentrated sulfuric acid, all of which were added into the digestion oven for digestion. The digestion was carried out at a temperature of 420 °C for about 1 h. The digestion solution was taken out once it became bright green and clear. After cooling, 50 mL of water was added slowly, and then an automatic Kjeldahl analyzer was used to determine the protein content in the sample. The fat content was determined by referring to the National Standard method GB/T 5009.6-2016 (Geber method) [37], and the lactose content was determined by referring to the National Standard method GB/T 5413.5-2010 (Rein-Enon method) [38].

### 3.5. Data Processing

#### 3.5.1. Two-Dimensional Correlation Spectra

The traditional one-dimensional Vis/NIR spectra take the wavelength or wavenumber as the horizontal coordinate and the absorbance as the vertical coordinate to characterize the spectral properties of the samples measured. Milk is a complex mixed system, and the interactions between light and milk are extremely complex [24]. Due to the serious overlapping of the Vis/NIR spectra from milk components, it is difficult to extract the feature information effectively in the traditional one-dimensional spectra. Two-dimensional correlation spectra provide the correlation information among the absorption peaks of the functional groups of different chemical components in the studied system, and can effectively analyze the weak peaks, overlapping peaks, and offset peaks with high spectral resolution [39].

Two-dimensional correlation spectra include synchronous and asynchronous spectra. The synchronous 2D correlation spectra are symmetric about the diagonal, showing the synergy between two dynamic signals. The peaks located on the diagonal are the autocorrelation peaks, the sizes of which indicate the total degree of the dynamic fluctuation of the spectral intensity in the correlation cycle. The peaks located outside the diagonal are the synchronous cross peaks, which indicate the synchronous change of the spectral signals at different wavelengths. When the spectra are affected by external disturbances, if the two spectral intensities increase or decrease at the same time, it is a positive correlation peak; however, if the two spectral intensities change in opposite directions, it is a negative correlation peak. The asynchronous 2D correlation spectra are antisymmetric about the diagonal, showing the order of intensity changes in the two dynamic signals. The specific theory can refer to the literature reported by Park et al. [40].

In this study, the spectra of fresh milk were averaged as the reference spectra. Firstly, the Vis/NIR spectra of milk and the reference spectra were subjected to a 2D correlation operation to obtain the synchronous 2D correlation spectral matrix of milk. Then, the autocorrelation spectral data of the synchronous 2D correlation spectral matrix was extracted. Finally, using the synchronous spectra of milk samples as a data set, the relationship between the features of synchronous 2D correlation spectra and the changes in milk freshness was analyzed, and classification models were constructed by combining LDA and SVM. All calculation and plotting of the 2D correlation spectra were accomplished using MATLAB 2018a.

#### 3.5.2. LDA Algorithm

The LDA algorithm is a classification method in which n-dimensional feature vectors (or samples) are linearly transformed into m-dimensional space (*m* < *n*) so that samples belonging to the same class are close to each other and samples of different classes are far apart [41]. Specifically, LDA seeks an optimal projective transformation matrix *V*.

(1) $$\underset{V}{max}\frac{\mathrm{tr}(V^{T}S_{B}V)}{\mathrm{tr}(V^{T}S_{W}V)}$$

where the inter-class dispersion *S_B_* and intra-class dispersion *S_W_* are defined as:

(2) $$S_{B}=\frac{1}{N}\sum_{i=1}^{C}N_{i}\left({\overset{-}{x}}_{i}-\overset{-}{x}\right){\left({\overset{-}{x}}_{i}-\overset{-}{x}\right)}^{T}$$

(3) $$S_{W}=\frac{1}{N}\sum_{i=1}^{C}\sum_{j=1}^{N_{i}}\left({\overset{-}{x}}_{ij}-{\overset{-}{x}}_{i}\right){\left({\overset{-}{x}}_{ij}-{\overset{-}{x}}_{i}\right)}^{T}$$

where *N* is the total number of samples, *N_i_* is the number of samples in the *i*th class, *C* is the number of class, ${\overset{-}{x}}_{i}$ is the average spectrum of samples in the *i*th class, ${\overset{-}{x}}_{ij}$ is the spectrum of *j*th samples in the *i*th class, and $\overset{-}{x}$ is the average spectrum of all samples.

#### 3.5.3. SVM

SVM is a pattern recognition method widely used in data mining applications to solve small-samples, nonlinear, and high-dimensional problems [42]. Like LDA, it provides a supervised classification method. SVM is used to map samples to a high-dimensional feature space using kernel functions. To find the optimal hyperplane in the high-dimensional feature space, the structural risk minimization criterion was applied to solve nonlinear classification problems. The input feature dataset, kernel function, and kernel parameters are the keys to affecting the performance of the SVM-based model. SVM theory is described in detail in the literature reported by Cristianini and Shawe-Taylor [43] and Devos et al. [44].

In this study, the following parameters were evaluated: (1) the input feature dataset, including the Vis/NIR spectral (Vis/NIR) dataset of milk and the 2D-Vis/NIR correlation spectral dataset; (2) the kernel functions, which were preferred between linear, polynomial, radial basis function (RBF), and sigmoid; and (3) the kernel parameters, in which the optimal parameters of different kernel functions were determined by grid search and particle swarm optimization algorithms.

The model performance was evaluated using accuracy, which is the ratio of correctly classified samples, and its expression was as follows:

(4) $${Accuracy}_{total}=\frac{V_{A}+V_{B}+V_{C}}{N_{A}+N_{B}+N_{C}}$$

(5) $${Accuracy}_{A}=\frac{V_{A}}{N_{A}},\;{Accuracy}_{B}=\frac{V_{B}}{N_{B}},\;{Accuracy}_{C}=\frac{V_{C}}{N_{C}}$$

where *V_A_*, *V_B_*, and *V_C_* are the number of correctly classified fresh milk (A), sub-fresh milk (B), and spoiled milk (C) samples; *N_A_*, *N_B_*, and *N_C_* are the number of fresh milk (A), sub-fresh milk (B), and spoiled milk (C) samples.

## 4. Conclusions

Spectroscopy as a rapid non-destructive detection method has promising applications in the analysis of dairy products. To effectively extract the useful information in spectra, the 2D correlation spectroscopy technique was introduced for the freshness analysis of milk, coupled with chemometric methods. Compared with Vis/NIR spectra, 2D-Vis/NIR spectra had the improved differentiation in different freshness levels of milk. Compared with the threshold-value-based models which could not completely identify the spoiled milk, LDA-based and SVM-based models had good modeling performances, with the latter being better. The SVM-based model using 2D-Vis/NIR spectra was proposed to identify the freshness of milk, with an accuracy of >97%. Compared with the SVM-based models using Vis/NIR data, the SVM-based models using 2D-Vis/NIR data had >15% higher prediction accuracy. 2D-Vis/NIR spectroscopy can more comprehensively and reliably reflect the variation of the internal quality of milk and achieve a rapid discrimination of milk freshness. Compared with traditional laboratory analysis, this method has rapid, non-destructive, and efficient advantages, and provides a technical support for the quality and safety tests of dairy products. In addition, although the prediction performance of the proposed model is good, the model needs to be updated with new samples, considering that milk quality is susceptible to external factors, such as the environment and temperature. It is expected that this technique will be transformed from laboratory research to commercial application in the future.

## Figures and Tables

**Table 1:** Changes of quality indicators in milk during storage.

Indicators	Range of Variation	Mean	Standard Deviation	CV ^1^	GB 19301-2010
Fat (%)	3.70~3.93	3.78	0.0711	0.0188	≥3.1
Protein (%)	3.72~3.76	3.74	0.0127	0.0034	≥2.8
Lactose (%)	5.03~5.35	5.15	0.1117	0.0217	- ^2^
Acidity (°T)	14.1~21.0	16.3	2.2904	0.1408	12~18
Relative density (20 °C/4 °C)	1.032~1.034	1.033	0.0007	0.0007	≥1.027

^1^ CV is the coefficient of variation. ^2^ “-” means not specified in GB 19301-2010.

**Table 2:** Prediction results based on Vis/NIR spectra and 2D-Vis/NIR spectral LDA models.

Data	Preprocessing	Calibration Set			Accuracy_total_ (%)	Prediction Set			Accuracy_total_ (%)
		A (%)	B (%)	C (%)		A (%)	B (%)	C (%)	
Vis/NIR	Raw spectra	96.3	90.7	96.3	93.8	100.0	93.3	77.8	90.9
	SNV	100.0	90.7	96.3	94.8	100.0	86.7	88.9	90.9
	MSC	100.0	97.7	100.0	99.0	100.0	86.7	88.9	90.9
	1st	100.0	90.7	100.0	95.9	0.0	100.0	0.0	45.5
2D-Vis/NIR	Raw spectra	100.0	95.3	100.0	97.9	100.0	86.7	100.0	93.9
	SNV	100.0	97.7	100.0	99.0	100.0	86.7	88.9	90.9
	MSC	100.0	97.7	100.0	99.0	100.0	86.7	100.0	93.9
	1st	100.0	97.7	77.8	92.8	100.0	0.0	0.0	27.3

**Table 3:** Prediction results of SVM-based models using Vis/NIR data or 2D-Vis/NIR data as calibration set.

Data Information	Indices	Linear			Polynomial			RBF			Sigmoid		
		A	B	C	A	B	C	A	B	C	A	B	C
Vis/NIR	SVs	18			21			24			20		
	A	27	0	0	27	0	0	27	0	0	27	0	0
	B	0	40	0	0	39	1	0	41	0	0	39	0
	C	0	3	27	0	4	26	0	2	27	0	4	27
	Accuracy (%)	96.9			94.8			97.9			95.9		
2D-Vis/NIR	SVs	10			7			6			6		
	A	27	0	0	27	0	0	27	0	0	27	0	0
	B	0	42	0	0	43	2	0	43	0	0	43	1
	C	0	1	27	0	0	25	0	0	27	0	0	26
	Accuracy (%)	99.0			97.9			100.0			99.0		

## Data Availability

The data used for the research described in this manuscript are available upon request.
